# Usefulness and Relevance of an eHealth Tool in Supporting the Self-Management of Chronic Obstructive Pulmonary Disease: Explorative Qualitative Study of a Cocreative Process

**DOI:** 10.2196/10801

**Published:** 2018-10-26

**Authors:** Malin Tistad, Sara Lundell, Maria Wiklund, André Nyberg, Åsa Holmner, Karin Wadell

**Affiliations:** 1 Unit of Physiotherapy Department of Community Medicine and Rehabilitation Umeå University Umeå Sweden; 2 School of Education, Health and Social Studies Dalarna University Falun Sweden; 3 Department of Radiation Sciences Umeå University Umeå Sweden; 4 Department of Public Health and Clinical Medicine Division of Medicine Umeå University Umeå Sweden

**Keywords:** COPD, eHealth, cocreation, self-management, primary care, chronic disease

## Abstract

**Background:**

New strategies are urgently needed to support self-management for people with chronic obstructive pulmonary disease (COPD) in primary care. The use of electronic health (eHealth) solutions is promising. However, there is a lack of knowledge about how such eHealth tools should be designed in order to be perceived as relevant and useful and meet the needs and expectations of the health professionals as well as people with COPD and their relatives.

**Objective:**

The objective of this study was to explore the aspects of an eHealth tool design and content that make it relevant and useful for supporting COPD-related self-management strategies from the perspective of health care professionals, people with COPD and their relatives, and external researchers.

**Methods:**

Data were collected during the development of an eHealth tool. A cocreation process was carried out with participants from two primary care units in northern Sweden and external researchers. Individual interviews were performed with health care professionals (n=13) as well as people with COPD (n=6) and their relatives (n=2), and focus group discussions (n=9) were held with all groups of participants. Data were analyzed using qualitative content analysis.

**Results:**

The overarching theme, *reinforcing existing support structures*, reflects participant views that the eHealth tool needs to be directly applicable and create a sense of commitment in users. Moreover, participants felt that the tool needs to fit with existing routines and contexts and preferably should not challenge existing hierarchies between health care professionals and people with COPD. Important content for health care professionals and people with COPD included knowledge about self-management strategies. Videos were regarded as the most effective method for communicating such knowledge.

**Conclusions:**

The cocreation in the development process enables participant perspectives and priorities to be built into the eHealth tool. This is assumed to contribute to a tool that is useful and relevant and, therefore, adopted into clinical practice and everyday life. Findings from this study can inform the development of eHealth tools for people with COPD in other contexts, as well as the development of eHealth tools for self-management support of other chronic diseases.

## Introduction

Pulmonary rehabilitation programs for people with chronic obstructive pulmonary disease (COPD) include exercise training and self-management strategies. These have been shown to decrease dyspnea; improve physical capacity, physical activity level, and health-related quality of life [1-4]; and be cost effective [5]. Self-management strategies include physical activity and appropriate food intake, recognizing and taking action if symptoms worsen, sputum evacuation, and breathing techniques. Each of these requires relevant knowledge and skills to be effective [1]. However, only a small proportion of people with COPD participate in pulmonary rehabilitation [6-9]. This may partly be due to insufficient adherence to nonpharmacological COPD guideline recommendations in primary care [10]. Furthermore, strenuous travel, exacerbation of symptoms, lack of motivation, and high costs have been reported as barriers to participation [6]. Since self-management is a core component of COPD management [1], a considerable proportion of people with COPD are at risk of insufficient access to support for these evidence-based interventions. Consequently, there is an urgent need to find new strategies to promote self-management support to people with COPD in primary care.

Electronic health (eHealth) includes digital technologies to inform, track, and monitor health in order to improve health and health services [11]. eHealth solutions have been suggested to have the potential to deliver support for self-management strategies to people with COPD [1,12], but the effectiveness and favorable features of such solutions remain to be determined. A recent meta-review of telehealth interventions to support self-management in COPD showed inconsistent effects [13]. In addition, recently published studies have report no difference in COPD-related health status after the use of a self-management platform [14] or the use of a system of monitoring and self-management support compared with usual care, apart from beneficial general health outcomes [15]. However, the functions and features of eHealth applications vary significantly and more research is needed.

Implementation of eHealth solutions has often proven to be challenging [16,17]. Implementation research concludes that the characteristics of the innovation to be implemented, the context, the recipients, and the method used for supporting the implementation influence whether the innovation is adopted [18]. In addition, studies have suggested that user involvement is important for understanding user needs, and it facilitates the use of eHealth solutions [16,19,20], whereas a lack of fit between users and the technology might hamper the adoption of technologies [17]. Both people with COPD and physiotherapists (PTs) have been shown to perceive an eHealth self-management application that intends to increase physical activity by goal-setting, advises on how to perform physical activity, and presents physical activity in steps to be stimulating and beneficial. However, PTs reported a low use of the eHealth application because of time constraints and costs [21]. More knowledge is needed about how eHealth tools should be designed to support the aspects of self-management other than physical activity that will meet the needs and expectations of health professionals and people with COPD and their relatives.

We decided to develop an eHealth tool in the form of an interactive website, the COPD web, directed toward two user groups—people with COPD and health care professionals providing primary care for these patients. The aims of the eHealth tool were to support people with COPD in their self-management strategies and facilitate the implementation of health care professionals' support for these strategies. To meet user needs and requests and contextual conditions while also following an evidence-based approach, we invited the user groups, that is, health care professionals and people with COPD and their relatives in primary care, as well as external researchers within the area of COPD to a cocreation process. The purpose of this study was to explore the aspects of the content and design of an eHealth tool that would make it relevant and useful for supporting COPD-related self-management strategies from the perspective of health care professionals, people with COPD and their relatives, and external researchers.

## Methods

### Study Design

**Figure 1:**
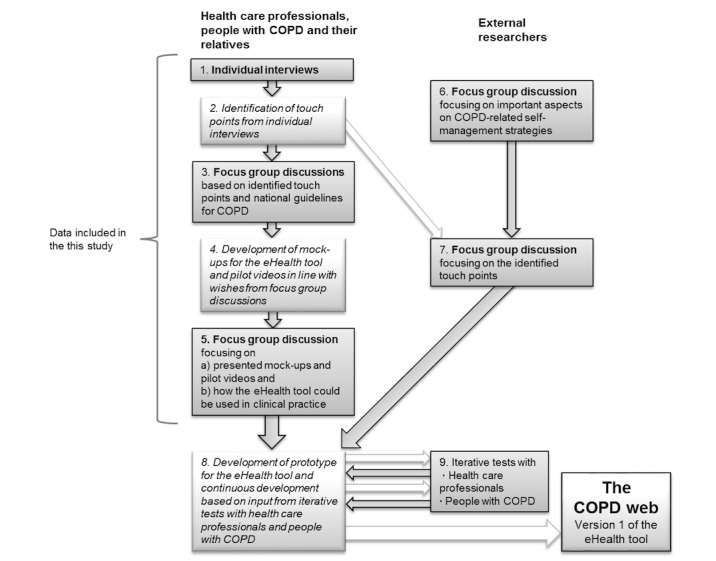
Structure of the development process of the eHealth tool. COPD: chronic obstructive pulmonary disease.

This explorative qualitative study is part of a larger research project based on cocreation and user involvement [22,23]. The study utilizes data from all of the individual interviews and focus group discussions carried out in the course of the development of an eHealth tool, the COPD web, aiming at supporting self-management strategies in people with COPD (Figure 1 and Table 1).

### Setting and Sample

Two primary care units in northern Sweden were invited to participate in the study, one situated in a city with a population of 120,000 inhabitants and one in a rural area with 2500 inhabitants. The urban primary care unit had about 7500 people enrolled and the rural unit had 2500 people. The primary care units provide outpatient care and, like almost all health care services in Sweden, are publicly funded.

The conditions for the use of eHealth solutions in Sweden in general are beneficial, and almost 100% of the population has access to the internet at home [24,25]. The possibility of reaching the older population is also relatively good as approximately 56% of those aged above 75 years use the internet [25].

### Recruitment of Participants

#### Participants for Individual Interviews

The nurses specialized in COPD care (henceforth denoted “COPD nurses”) at the primary care units were asked to participate in individual interviews. They were asked to suggest 1 or 2 additional nurses and physicians who met people with COPD in their clinical practice. Furthermore, all PTs, occupational therapists (OTs), dieticians, and medical social workers (MSWs) employed or engaged as consultants at these units were asked to participate. In total, 16 health care professionals were invited and 13 were finally included (Table 2). Due to very limited working time at the unit, illness, or no experience with COPD, 1 OT and 2 MSWs declined participation.

**Table 1:** Description of the components in the development process and data collection.

Component in the development process	Group of participants and number of individual interviews (n) or focus groups (FG)	Content
1. Individual interviews^a^	Health care professionals (n=13) People with COPD^b^ (n=6) / relatives (n=2)	Semistructured interviews with health care professionals and people with COPD and their relatives.
2. Identification of touch points from individual interviews	Intermediate work by the researchers	Identification of touch points (ie, topics that seemed crucial or were mentioned by several of the interviewees).
3. Focus group discussions^a^	Health care professionals (FG=2) People with COPD and their relatives (FG=2)	The identified touch points and self-management strategies that were highly prioritized in the National Guidelines for COPD were presented to the participants. The participants were encouraged to reflect on the topics that were presented and particularly on how an electronic health (eHealth) tool could facilitate provision of, or give support for, such self-management strategies.
4. Development of mock-ups for the eHealth tool and pilot videos in line with wishes from focus group discussions	Intermediate work by the researchers	Based on the wishes and needs expressed during the individual interviews and focus group discussions, mock-ups for the website and pilot videos were developed showing breathing techniques for stair climbing and muscle strength training.
5. Focus group discussions^a^	Health care professionals (FG=2) People with COPD and their relatives (FG=1)	The mock-ups and the pilot videos were presented. The participants were encouraged to reflect on the basic structure, the colors, wordings, and how well the pilot films served their purpose. Moreover, the participants were asked to reflect on how the website could be introduced to people with COPD and how the use of the website should be followed up.
6. Focus group discussions^a^	External researchers (FG=1)	Based on their scientific knowledge about COPD, the external researchers were encouraged to identify and reflect on important interventions and self-management strategies that would be important to include on the website.
7. Focus group discussions^a^	External researchers (FG=1)	A summary of the suggestions, wishes, and needs brought up by the health care professionals and people with COPD and their relatives were presented. The researchers were asked to reflect on how the interventions and self-management strategies should be presented considering both scientific correctness and the need to allow for adaptations to local conditions. Moreover, the researchers were asked to prioritize between the suggestions, wishes, and needs.
8. Development of prototype for the eHealth tool	Intermediate work by the researchers	A prototype for the eHealth tool was developed based on input from the individual interviews and focus group discussions. The iterative tests (9) led to further development.
9. Iterative tests	Health care professionals (n=6) People with COPD (n=6)	Iterative tests focusing on what words to use in the menu structure and the navigation of the website were performed.

^a^Data for this study was collected during this component.

^a^COPD: chronic obstructive pulmonary disease.

The COPD nurses at both units were also asked to assist in identifying 3 people with COPD—with variations in disease severity and sex—for participation in the individual interviews. A total of 6 people with COPD were invited, and all of them agreed to be interviewed (see Table 2). The people with COPD were asked to nominate a relative who the researchers could contact and ask for participation in the interviews. Accordingly, 3 relatives were asked and 2 agreed to participate (see Table 2).

#### Participants for Focus Groups

In order to avoid traveling of the participants, the focus groups (Table 3) were formed separately in urban and rural areas. Our intention was to include 1 COPD nurse, 1 PT, and 1 physician from the individual interviews at each unit in the focus groups for health care professionals. However, because the physicians were unable to participate due to time constraints, a district nurse with extensive experience in the care and support for people with other chronic diseases at the primary care unit and a physician with a special interest in COPD employed at another primary care unit joined one focus group each. Thus, one group consisted of 2 nurses and 1 PT, and the other group consisted of 1 COPD nurse, 1 PT, and 1 physician.

**Table 2:** Description of participants in the individual interviews.

Participants			Value
**Health care professionals**			
	Nurse, n		5
	Physician, n		3
	Physiotherapist, n		2
	Occupational therapist, n		1
	Dietician, n		2
	Professional experience (years), mean (range)		20 (3-31)
**People with chronic obstructive pulmonary disease**			
	**Sex, n**		
		Male	2
		Female	4
	Age (years), mean (range)		74 (65-80)
	FEV_1_%^a^predicted, mean (range)		58 (32-91)
**Relatives (roles), n**			
	Son or daughter		1
	Spouse		1
**External researchers**			
	Nurse, n		1
	Physician, n		1
	Physiotherapist, n		1
	Dietician, n		1
	Professional experience (years), mean (range)		24 (15-32)

^a^FEV_1_%: Forced expiratory volume in 1 second.

**Table 3:** Composition and number of participants in the focus groups.

Participants in the focus groups			n
**Health care professionals**			
	Focus group 1 (nurse, physician, and physiotherapist)	3	
	Focus group 2 (nurses and physiotherapist)	3	
**People with chronic obstructive disease and relatives**			
	Focus group 1	4	
	Focus group 2	3	
**External researchers**			
	Focus group 1 (nurse, physician, or physiotherapist and dieticians)	4	

The people with COPD and their relatives who had participated in the individual interviews were asked to partake in focus groups, among whom, 5 people with COPD and 2 relatives agreed. Thus, one group consisted of 2 individuals with COPD and 1 relative, but one of the individuals with COPD never turned up. The other group consisted of 3 individuals with COPD and 1 relative. Moreover, 4 external researchers—including a physician, a PT, a COPD nurse, and a dietician—who were engaged in both research and clinical practice within the field of COPD were invited to a separate focus group. All of the researchers agreed to participate.

**Table 4:** Theme, categories, subcategories, and groups of participants.

Theme, categories, and subcategories			Group of participants
**Reinforcing existing support structures**			
	**Supportive and noninterfering**		
		Handling the disease	People with chronic obstructive pulmonary disease (COPD) and their relatives
		Applying evidence-based care	Health care professionals Researchers
		Fitting into the current routines	Health care professionals People with COPD
		Keeping control	Health care professionals Researchers
	**Meaningful and urgent**		
		Visualized messages that enable self-identification	Health care professionals People with COPD and their relatives Researchers
		Easily accessible and distinct messages	Health care professionals People with COPD and their relatives Researchers
		Creating engagement	Health care professionals People with COPD Researchers

### Process of Data Generation and Cocreation

All individual interviews and focus group discussions were carried out between January and May 2015 as part of the development of the eHealth tool (Figure 1 and Table 1). One of the authors (MT) performed the individual interviews with health care professionals, and 2 of the authors (MT and SL) performed the interviews with the people with COPD and their relatives. Health care professionals were interviewed at their work places with the exception of one interview performed at a restaurant. The interviews with people with COPD and their relatives were performed at their homes (n=3), at the university (n=3), at a restaurant (n=1), and at their primary care unit (n=1) in accordance with their wishes. The interviews lasted between 30 and 60 minutes. MT moderated the focus group discussions with people with COPD and health care professionals with support from SL and KW, who raised follow-up questions and added reflections. SL or MT moderated the focus group discussions with the external researchers with support from KW. All of the focus group discussions lasted approximately 1 hour. Interviews and focus group discussions were audiorecorded and transcribed verbatim by a professional transcriber, and the transcripts constitute the data for this study. All data were continuously analyzed during the development of the prototype for the eHealth tool. For this study, we accumulated all of the data in order to summarize and deepen the analyses.

### Analysis

The transcribed interviews were analyzed using qualitative content analysis [26]. Initially, the transcripts were read through in order to get a “sense of the whole” [26]. In the next step, all data derived from the individual interviews and focus groups with health care professionals were inductively coded using software Open Code 4 [27]. Codes with similar content were grouped into subcategories that were abstracted into higher-order categories. Thereafter, the same process was carried out with all of the data derived from the individual interviews and focus groups with people with COPD and their relatives and with external researchers, separately. To complete the analysis, categories and subcategories from the different groups of participants were collated at a higher interpretive level, and after discussions and reflections, the authors agreed on a set of 7 subcategories, 2 categories, and 1 theme (Table 4). The analysis was performed by MT in close collaboration with SL and involved continuously going back and forth between the whole empirical data and parts thereof. Credibility was strived for through recurrent triangulation between all of the authors with various competencies and perspectives regarding the most credible analysis and interpretation of the findings [26].

### Ethics

Approval was granted by the regional ethical review board of Umeå, Sweden (Dnr 2014/319-31). Written informed consent was given by all participants, and their confidentiality was ensured throughout the whole research process, including the storage, publication, and dissemination of results.

## Results

### Reinforcing Existing Support Structures

The analysis resulted in the theme *reinforcing existing support structures*, which, together with the interrelated categories and subcategories (Table 4), represents the participants’ overall view on how an eHealth tool could have the potential to improve existing support for self-management. It was seen as being able to reinforce the information and interventions from the health care professionals and could provide easier access to information and support for people with COPD and their relatives. All of the involved groups emphasized that the content should be directly applicable and must create engagement among its users. Moreover, they emphasized that the eHealth tool should fit with existing routines and contexts and preferably not challenge existing hierarchies between health care professionals and people with COPD.

### Supportive and Noninterfering

The category supportive and noninterfering refers to the content of the eHealth tool that focuses on the practical and concrete level in the management of COPD. For people with COPD, this meant content linked to everyday challenges that could decrease the consequences of the disease in daily life. For the health care professionals and researchers, this meant a tool that could support patients’ self-management and increase their readiness to act as well as support health care professionals’ knowledge and way of working while fitting into their prevailing routines.

#### Handling the Disease

People with COPD described a responsibility for handling the disease, and they perceived pressure to stay physically active, to do breathing exercises, or to quit smoking. A common view was that COPD was a disease that was ignored by physicians and the entire health care system. Furthermore, with the exception of smoking cessation, nonmedical issues were not viewed as something you should “bother” the busy primary care with. Because relatives were not always involved, expressed as “COPD is nothing you talk to relatives about,” the responsibility for patients’ body and lifestyle choices was foremost perceived as their own:

Because I’ve had COPD for many years, and no one cares. But I have a responsibility to my own body—a great responsibility in order for me to be able to survive. And I don't want to become this big lump who just lies on the floor…so I just have to get myself out of the house…

Participant with COPD

At the same time, the people with COPD had only limited knowledge about the disease and self-management. Furthermore, they had scarce knowledge about what kinds of support were available through health care services or when to contact the primary care. Therefore, the eHealth tool could, according to both people with COPD and their relatives, contribute valuable information and deeper understanding about, for example, exacerbations, nutrition, or strategies for sputum evacuation. The eHealth tool was also considered to have the potential to support exercise training by providing videos of exercises suitable for a home environment for people who were motivated because training at a gym was expensive and might require strenuous travel. A possibility to send questions to the COPD nurse through the eHealth tool and have them answered was raised as a suggestion.

Handling the disease in everyday life also involved feelings of self-blame and worthlessness as well as hiding the self-inflicted disease by saying things like “I am just a bit out of breath” instead of naming the disease. The eHealth tool was seen as a tool that could deal with the urgent “blame-yourself question,” and one suggested strategy for doing that was to produce short videos of critical situations such as getting the diagnosis or chatting about the disease with friends.

#### Applying Evidence-Based Care

The subcategory applying evidence-based care captures the views of the role an eHealth tool could play in supporting the application of guideline recommendations and evidence in clinical practice. Health care professionals suggested that the eHealth tool could offer knowledge and support for self-management strategies in order to meet their needs for knowledge. They expressed great variability in their COPD-related knowledge, and while some perceived a need for very basic knowledge, others expressed a need for knowledge related to their own professional practice. For example, the PTs who primarily catered to patients with musculoskeletal disability in their daily practice expressed needs for knowledge about breathing techniques and about how much one could “dare to push them” during physical training. Moreover, easy access to screening tools, material for patient education, and updated information about local exercise groups was highly desirable.

The eHealth tool was considered by both researchers and health care professionals to have the potential to support people with COPD in self-management strategies and to strengthen their ability to influence their health, interpret symptoms, and take relevant actions such as contacting the health care system. Portraying people with COPD who had succeeded in, for example, increasing their level of physical activity as role models on the eHealth tool was thought to support other patients in their use of self-management strategies. A common view was that people with COPD are a low-powered group that neglects important symptoms such as weight loss and symptoms indicating an exacerbation of their disease. However, as people with COPD might be “stigmatized and depressed and feel bad” and have bad experiences from previous contacts with health care services, the researchers also acknowledged that they might find it difficult to ask for services.

A crucial issue in the researchers’ discussion was how the newly published, evidence-based National Guidelines for COPD care [28] and other evidence should be applied in primary care. The eHealth tool could, for instance, provide concrete advice on how people with COPD could start increasing their level of physical activity and how health care professionals could use the recommended screening tools and interpret the results in order to identify patients with the greatest needs. Furthermore, questions related to how the guideline recommendations could be adapted to clinical contexts and how this was described on the eHealth tool were seen as essential. This can be exemplified through a discussion among the researchers related to the 6-minute walking test, which is highly prioritized in the national guidelines but requires a 30-meter corridor in order for cut-off values to be valid.

But I still think that we need to come out with the recommendation that if you only have ten meters, then that’s what you should use to do it. If you then do it the same way every time.

External researcher

Another issue that might demand contextual adaptation, raised by the researchers, was how work was organized. Contributions from the eHealth tool could be to describe what interprofessional collaboration and evidence-based practice included but not to define “who should do what.”

#### Fitting Into the Current Routines

The subcategory fitting into the current routines reflects the participants’ view that the eHealth tool had to fit the contextual conditions in the primary care and the habits and interest of people with COPD in order to be used regularly. The health care professionals pointed to the dilemma that the use of a website would require access to computers in a way that was not in concordance with the present situation. Flexible use of the eHealth tool without being tied to a desktop seemed helpful, and wishes to “have an iPad in my room” were expressed. Time was another resource that was emphasized because the introduction of the eHealth tool might require longer visits.

Furthermore, a challenge related to the use of the eHealth tool was variation in interest, motivation, and computer skill among the people with COPD. Even though almost all the people with COPD and their relatives owned a computer, some experienced a lack of knowledge about how to use it, as well as a lack of interest. The use of computers could be associated with previous work, and one relative had made a promise “to never sit by the computer when retired.”

Regarding an eHealth tool as support for exercise training, a common view was that participating in a group together with other people with COPD for exercise training seemed more fun compared with doing exercises at home. Doing exercises at home was considered to require strong motivation, and participating in a group and having an inspiring instructor was seen as the best support for physical exercise. Limited opportunities to participate in such groups in the rural area was also put forward.

#### Keeping Control

Even though the health professionals’ and researchers’ ambition to strengthen the patients was prominent, the subcategory keeping control captures how the eHealth tool could potentially challenge the well-established hierarchy between health care professionals and patients. A few thoughts were brought up among them, suggesting that patients could be unable to handle all of the information and that patients who were too knowledgeable might induce a risk of “being questioned.” Therefore, it was suggested that the patients should not be able to access information primarily directed to the health care professionals on the eHealth tool, such as how to organize team-based care and alternative interpretations of symptoms. Furthermore, encouraging people with COPD to ask for specific health services, such as support for physical exercise, was not always appreciated because the primary care unit’s right to prioritize the services offered was considered important.

No one else should get involved. Because that’s how the financial conditions are. So I don't think you should promise [on the eHealth tool] that someone else will do something.

Health care professional

Furthermore, the national guidelines were seen as tools for health care professionals that were difficult to communicate to the public.

### Meaningful and Urgent

The category meaningful and urgent reflects the participants’ perspective that the eHealth tool should be designed so that it speaks distinctly and directly to its target groups. A straightforward message and wording that included all groups of health care professionals was seen as crucial in order to promote its use.

#### Visualized Messages That Enable Self-Identification

All groups of participants viewed visualized messages that enable self-identification on the eHealth tool as an advantageous way to communicate information, instructions, and advice. People with COPD and their relatives perceived that videos would be “more efficient” and “informative” compared with text or instructions on paper. The health care professionals suggested several issues that could be communicated through videos such as the handling of positive expiratory pressure devices and energy conservation techniques. To make the messages meaningful, people with COPD suggested that the videos should allow them to identify themselves with the people in the videos. This could be done by showing people with COPD instead of actors and by including “young, old, white, and black people; persons with disabilities; and those who are able-bodied.” In order to further enable identification, the health care professionals put forward that both positive and negative experiences of using self-management strategies, as well as different stages of the disease, could be represented in videos.

#### Easily Accessible and Distinct Messages

The importance of communicating easily accessible and distinct messages on the eHealth tool with a focus on short bits of information written in an “understandable language” was brought up by all groups. Health care professionals, people with COPD, and their relatives emphasized that the eHealth tool should be easy to find on the internet, that the written information should be illustrated with pictures, that one should be able to listen instead of having to read, and that the information should be printable. When pilot videos were shown during the focus group discussions, both health care professionals and people with COPD pointed out the importance of instructions that specified the purpose and benefits of, for example, breathing techniques and physical exercises.

Either I was very inattentive…but the instructions…well, I understood what to do with the rubber band and all that, but what's the point of it?

Participant with COPD

Health care professionals also thought that the eHealth tool would be accessed to a greater extent if registration and log-in could be avoided or at least be voluntary.

#### Creating Engagement

The subcategory creating engagement captures the participants’ view that the eHealth tool would need to arouse interest among its potential users, which involves both aspects of the content and the introduction of the tool. The choice of wording was thought to influence health care professionals’ motivation to use the eHealth tool, and the researchers suggested that the expression “pulmonary rehabilitation” was not the most suitable in order to engage all groups of health care professionals.

Rehabilitation is so focused on physiotherapy. But if you call it ‘health-promotion,’ then it includes, like, all of the professions in this line of work. It supports interprofessional collaboration.

Researcher

The people with COPD perceived that a face-to-face introduction, preferably by the COPD nurse, would be most advantageous. Some type of written information was considered unavoidable, even though “being flooded by leaflets” was a common experience, and a small card with the address to the website or a leaflet was preferred. Health care professionals suggested printed material with information about the eHealth tool to hand over to both people with COPD and their relatives in order to involve them as well.

Because many people with COPD also suffered from comorbidities, it was considered important to meet the needs of a specific patient in order to create engagement and make the eHealth tool relevant.

When you have COPD, you often have many other illnesses too, and do you take those into account? Well, the patient certainly asks himself that “But I have heart failure, too. Or diabetes, or…”…When you’re supposed to do what they say in this video. It just isn’t accurate. Click. Delete. And then you forget the video

Health care professional

Individualization was considered to be possible if information and videos on the eHealth tool targeted different stages of the disease. Health care professionals then could pick information considered relevant for a specific individual during the introduction of the tool.

## Discussion

### Principal Findings

The number of eHealth solutions that are being developed has increased rapidly in recent years. In order to enable implementation, it is important that the development of such solutions is informed by the needs and preferences of the potential users and by contextual conditions [16,17,19,20]. Accordingly, data for this study were collected during the cocreation process of an eHealth tool aimed at supporting self-management strategies in people with COPD. Key findings, reflecting study participants’ perspectives and captured in the theme *reinforcing existing support structures*, suggest that an eHealth tool aiming to support self-management strategies should facilitate the adaptation of guideline recommendations and evidence into everyday practice. Furthermore, the eHealth tool should reflect the urgency of self-management issues and communicate this in a distinct message while fitting into the existing routines and not threating the existing hierarchy between health care professionals and patients.

### Interpretation of Findings

Insufficient knowledge about how to apply guideline recommendations and other evidence-based interventions in primary care was described by health care professionals, and similar findings have also been reported in previous research [10,23,29,30]. Insufficient knowledge has also been reported as a barrier to guideline adherence in COPD care [10,23,29,31]. Furthermore, having a thorough understanding of what a new practice entails and the relevant skills has been described as crucial for the successful adoption of a new practice [18,32-34]. Consequently, as captured in the subcategory *applying evidence-based care*, the study participants emphasized that an eHealth tool should provide concrete examples and suggestions on how to adapt and apply guideline recommendations in order to facilitate evidence-based practice. An eHealth tool alone cannot be expected to make up for insufficient knowledge and skill, but it might have the potential to facilitate an implementation process.

The eHealth tool was considered to have the potential to strengthen the people with COPD and increase their readiness to act and to be more involved in their own care. The emphasis on patients’ involvement is in line with the national and international development toward person-centered health care systems [35,36] that include sharing of information and knowledge in order to create a common understanding and to build a partnership between patients and health care professionals [37-39]. On a national level, efforts that help patients become experts on their conditions are imperative and have been called for by the Swedish authorities [40]. However, as illustrated in the subcategory *keeping control*, patients taking up the role of experts—who ask for services and interpret their own symptoms—might be perceived as a challenge to the health care professionals’ authority. This is supported by a previous review in which an unwillingness and reluctance to encourage patient participation and to delegate power to patients was reported [41], and limiting the amount of information given to patients was one way of maintaining control. In the context of COPD, a study of health care professionals involved in providing pulmonary rehabilitation ranked the importance of patients’ adherence to medical advice considerably higher than having the patient involved as a team member or having the patient be an independent information seeker [42]. Even though most health care professionals seem to welcome more active and involved patients, the fact that not everyone embraces this shift in the patient’s role must be acknowledged and challenged.

An important finding is that people with COPD only turned to primary care when faced with strictly medical issues and not issues related to self-management. One explanation for this, supported by previous research, is insufficient knowledge about self-management [43,44], including insufficient knowledge about what services and support are available from primary care. Another explanation might be the experience of guilt and shame associated with a self-inflicted disease that was described by the people with COPD in this study and also reported in other studies [45-48]. Such feelings might lead to a situation where patients distance themselves from their symptoms and minimize their needs, thus, avoiding seeking advice and instead adapting to a life with unnecessary disabilities [46,48]. Because self-management plays a prominent role in the treatment of COPD, there is an obvious need to provide easily accessible support for self-management, including information on when to contact primary care and information on what support might be available. The internet and eHealth solutions seem to be appreciated and valued sources of information and support for people with COPD [47,49], and consequently, it is important that such support, based on the needs and wishes expressed in this and similar studies, is available.

In this study, videos were suggested as important measures for communicating the self-management interventions as well as for addressing questions about the shame associated with a self-inflicted disease. Previously reported eHealth interventions have involved persuasive technologies such as remote monitoring of physical activity [21] and self-monitoring of health values [50], but no such components were suggested by the people with COPD in our study. However, the absence of such proposals and desires is hardly surprising as it might be necessary to have knowledge about such interventions in order to propose them. The use of videos for demonstration of self-management intervention is in accordance with “modeling,” which is one of the ingredients described to enhance self-efficacy for self-management in chronic conditions [1,51]. Modeling can be accomplished through the use of videos or pictures that reflect the population of concern [51] and might thereby have the potential to influence people’s behavior. However, the use of only videos and written information as methods for supporting self-management strategies on an eHealth tool might be insufficient, and additional persuasive technologies might be needed in order to promote behavior change.

### Strengths and Limitations

In the research process toward an eHealth tool for enhanced self-management, a major strength of this study is its focus on user involvement and cocreation. Trustworthiness has been strived for by involving health care professionals representing different professions, people with COPD and their relatives, and external researchers, who have provided several perspectives on the relevance and usefulness of the eHealth tool. Furthermore, the fact that the sample included both rural and urban areas and people with COPD at different stages of their disease is essential because the perceived needs and relevance for eHealth solutions might differ based on the distance from health care services and severity of the disease. The authors’ broad range of competencies and perspectives, and recurrent reflection during the process of analysis, further added to the trustworthiness. The limited number of people with COPD and their relatives in the study must be considered a weakness as this might have limited the variation in the findings. Furthermore, a greater representation of physicians in the health care professionals’ focus groups, as well as representation of OTs, dieticians, and MSWs, would have been beneficial. However, as the findings represent a broad range of experiences from 3 groups of participants, we assume that the results could be generalized to similar health care contexts.

### Conclusions

Self-management is an ongoing and never-ending task for many people with chronic diseases, and the development of tools that are accessible and meet the needs of the users, including both health care professionals and patients, is imperative. The findings of this study, such as the need for knowledge about how to apply guideline recommendations, the need for more knowledge among people with COPD, how to create engagement among the users, and eHealth tools as potential threats to hierarchies, are presumably generic and can inform the development of eHealth tools for self-management support in other chronic diseases. The involvement of the user groups and the careful analysis of their views and perceptions enable their perspectives and priorities to be built into the eHealth tool and will most likely contribute to a tool that has the potential to be adopted in clinical practice and in everyday life.

## Abbreviations

COPD: chronic obstructive pulmonary disease
eHealth: electronic health
MSW: medical social worker
OT: occupational therapist
PT: physiotherapist
